# Overview and Analysis of the Cost of Drug Programs in Poland: Public Payer Expenditures and Coverage of Cancer and Non-Neoplastic Diseases Related Drug Therapies from 2015–2018 Years

**DOI:** 10.3389/fphar.2020.01123

**Published:** 2020-08-14

**Authors:** Aneta Mela, Łukasz A. Poniatowski, Bartłomiej Drop, Marzena Furtak-Niczyporuk, Janusz Jaroszyński, Witold Wrona, Anna Staniszewska, Jan Dąbrowski, Anna Czajka, Beata Jagielska, Monika Wojciechowska, Maciej Niewada

**Affiliations:** ^1^ Department of Experimental and Clinical Pharmacology, Centre for Preclinical Research and Technology (CePT), Medical University of Warsaw, Warsaw, Poland; ^2^ Department of Neurosurgery, Maria Skłodowska-Curie Memorial Cancer Center and Institute of Oncology, Warsaw, Poland; ^3^ Department of Information Technology and Medical Statistics, Faculty of Health Sciences, Medical University of Lublin, Lublin, Poland; ^4^ Department of Public Health, Faculty of Medicine, Medical University of Lublin, Lublin, Poland; ^5^ Chair of Administrative Procedure, Faculty of Law and Administration, Maria Curie-Skłodowska University of Lublin, Lublin, Poland; ^6^ HealthQuest Sp. z o.o., Warsaw, Poland; ^7^ Department of Pharmacology, National Medicines Institute, Warsaw, Poland; ^8^ Department of Neurology, Czerniakowski Hospital, Warsaw, Poland; ^9^ Department of Oncological Diagnostics, Cardiooncology and Palliative Medicine, Maria Skłodowska-Curie Memorial Cancer Center and Institute of Oncology, Warsaw, Poland; ^10^ Department of Pediatric Nephrology, Medical University of Lublin, Lublin, Poland

**Keywords:** drug programmes, public payer expenditures, reimbursement, cancer drugs, drug therapy, hospital medicinal products, economic burden

## Abstract

**Background:**

In Poland drug programmes developed by the Minister of Health and financed by the National Health Fund are special reimbursement frameworks of innovative, expensive, and mostly hospital based medical products used for a small number of patients.

**Research Design:**

The research presented in this paper is based on data analysis published by the National Health Fund in Poland. The analysis focused on estimating public payer expenditure on drugs available within drug programmes from 2015 to 2018.

**Results:**

In subsequent years, reimbursement of drugs used within drug programmes was associated with the National Health Fund budget expenditure of 635 mln USD, 755 mln USD, 854 mln USD, and 921 mln USD, respectively. Reimbursement of oncology drug programmes constituted 48.1%, 42.5%, 47.1%, and 52.4% and were approximately 305, 312, 402, 483 mln USD, whereas values of non-oncology drug programmes were approximately 330, 434, 452, and 438 mln USD which constituted 51.9%, 57.5%, 52.9%, and 47.6% respectively.

**Conclusion:**

Despite the fact that the expenditure on drug programs in Poland are increasing every year, they undoubtedly improve the patient’s access to the most innovative oncological and nononcological therapies in the Polish healthcare system.

## Introduction

A drug reimbursement system in Poland is the constitutional response for providing access to health services by the country. In fact, contemporary medicine may not operate without drug ordinance. However, drug prices may be an insurmountable obstacle for many patients, therefore reducing access to health services. The entitlement of a patient-beneficiary to guarantee services financed (cofinanced) from public funds entails, among others, a right to drugs. An act on reimbursement of drugs is therefore an attempt to realize a duty incumbent on a country to guarantee patients their constitutional right (Kaczmarczyk, 2015).

Poland is an example of a country where medicines cannot be reimbursed without formal procedures. It is not possible for a drug to be reimbursed for a new medical indication without an assessment of health technologies. This concerns medicines that are available to patients as part of drug programs.

Following the example of other developed countries, in 2005, Poland introduced a health technology assessment system to the drug reimbursement process by establishing an advisory body for the Minister of Health – The Agency for Polish Health Technology Assessment (AHTAPol), whose tasks include in particular, developing recommendations regarding the financing of health technologies.

The applicant, who is most often a pharmaceutical company, applies for the reimbursement through the Minister of Health, who instructs AHTAPol to perform a verification analysis. In accordance with the guidelines of the president of AOTMiT, the drug manufacturer, in addition to analyzing the decision problem, has to prepare three types of analyses: the clinical effectiveness analysis, the economic analysis and the analysis of the impact on the healthcare system. The analysis of clinical effectiveness addresses the question of what additional clinical benefits, in relation to the currently used standard in the given indication, will be brought by the introduction of the new technology. The second analysis assesses the cost-effectiveness of the treatment. The Polish legislator specifies situations in which the reimbursement of a new therapy would be cost-effective relative to the alternative form of treatment. This happens when the so-called cost-effectiveness ratio does not exceed the profitability threshold, which is set at three times the GDP per capita/QALY (quality-adjusted life-year). The last of the indicated analyses concerns the assessment of the impact of introducing a new medical technology on the payer’s budget. It allows for the determination of the additional expenses the payer will have to incur as a consequence of introducing a new therapy into clinical practice.

AHTAPol’s advisory body is the Transparency Council that issues its opinion based on the verification analysis. AHTAPol’s final position is the president’s recommendation, which is one of the elements that influences the final reimbursement decision made by the Minister of Health. It is worth noting that in Poland, the Minister’s decision does not have to coincide with the president’s recommendation, which happens quite often in practice.

The entity financing healthcare services in Poland is the National Health Fund (“NFZ”), subject to the Minister of Health. The regulations provide a total budget for reimbursement of no more than 17% of the sum of public funds allocated for the financing of guaranteed services in the NFZ financial plan. In order to protect this budget and reduce public expenditure on reimbursement, instruments such as the pay back obligation, risk sharing instruments and others have been introduced.

In Poland, reimbursement from public funds applies to drugs, foodstuffs for particular nutritional use, and medical devices in the range of registered indications or in the indication specified by the clinical state. These are available on the market and have the identification code – EAN (European Article Number) or other code equivalent to EAN (Jahnz-Różyk et al., 2017). The list of reimbursed drugs is updated every two months and priority for reimbursement is granted to drugs providing the greatest therapeutic outcomes at the lowest cost (Lipska et al., 2017).

Regulations allow the following categories of reimbursement access:

- Products available for purchase in the pharmacy with a prescription (in the whole range of registered indications and purposes or in an indication specified by the clinical state);
- Products used within chemotherapy (in the whole range of registered indications or in an indication specified by the clinical state);
- Products covered within specific framework called drug programmes (Ustawa z dnia 12 maja, 2011; Badora et al., 2017).

Drug programmes are developed by the Minister of Health, however, the entity responsible for implementation, financing, monitoring, surveillance, and control of the drug programmes is the National Health Fund. Legal basis regulating implementation of drug programmes is regulation number 125/2017/DGL of the President of the National Health Fund on requirements for concluding and realising agreements in type: hospital treatment in a range, drug programmes issued on 19 December 2017 (Ustawa z dnia 27 sierpnia, 2004; Załączniki nr 4 do zarządzenia Nr 125/2017/DGL Prezesa Narodowego Funduszu Zdrowia z dnia 19 grudnia, 2017).

Drug programmes play a significant role in therapeutic programmes, ensuring patients access to modern treatment methods. These programmes comprise very expensive drug technologies used in a small number of patients. Essentially, drug programmes are dedicated to patients in whom previous treatment did not provide the desired outcome, but also to those with rare and ultra-rare diseases. Drug programmes involve treatment of oncological diseases (e.g., breast cancer, kidney cancer, malignant lymphomas, melanoma, etc.) and nononcological diseases (e.g., hepatitis B and C, multiple sclerosis, rheumatoid and psoriatic arthritis etc.) (Załączniki nr 4 do zarządzenia Nr 125/2017/DGL Prezesa Narodowego Funduszu Zdrowia z dnia 19 grudnia, 2017).

Drugs used within drug programmes were enumerated in the catalogue of reimbursed drugs issued by the Minister of Health based on article 37 section 1 of the Act from 12 May 2011 (Ustawa z dnia 27 sierpnia, 2004). The catalogue contains information on active substances, the route of administration, size, unit, name, form, and dose (Ustawa z dnia 6 września, 2001; Ustawa z dnia 6 września, 2001; Ustawa z dnia 12 maja, 2011; Załączniki nr 1 do zarządzenia Nr 125/2017/DGL Prezesa Narodowego Funduszu Zdrowia z dnia 19 grudnia, 2017). Particular drugs might be simultaneously listed within the drug programme and chemotherapy catalogue but in separate indications.

Eligible patients must meet the criteria, included in the programme description, for entry into the drug programme (Załączniki nr 6-16 do zarządzenia Nr 125/2017/DGL Prezesa Narodowego Funduszu Zdrowia z dnia 19 grudnia, 2017). The drug programme description involves the criteria for patient enrolment and exclusion from treatment, drug dosing, administration method, and a list of diagnostic tests to be performed during qualification for the programme and necessary for treatment monitoring (Załącznik nr 17 do zarządzenia Nr 125/2017/DGL Prezesa Narodowego Funduszu Zdrowia z dnia 19 grudnia, 2017). A patient’s participation in a drug programme entails, on the one hand, receiving innovative drugs, and on the other hand, complete adherence and timely participation in control tests.

Healthcare services under drug programmes are separately contracted by the NHF. By way of public tender or prognosis providers for realisation of drug programmes within a voivodeship, a group of hospital centres are selected (§3. pkt 1 zarządzenia Nr 125/2017/DGL Prezesa Narodowego Funduszu Zdrowia z dnia 19 grudnia, 2017; Załączniki nr 3 do zarządzenia Nr 125/2017/DGL Prezesa Narodowego Funduszu Zdrowia z dnia 19 grudnia, 2017). Realisation of healthcare services within drug programmes may be conducted outpatient, in one day treatment, hospitalisation, and at home. Treatment mode depends on disease type, treatment purpose, drug form, time needed for drug administration, and necessary monitoring after drug administration in terms of possible outcomes or side effects (Załączniki nr 2 do zarządzenia Nr 125/2017/DGL Prezesa Narodowego Funduszu Zdrowia z dnia 19 grudnia, 2017; §8 zarządzenia Nr 125/2017/DGL Prezesa Narodowego Funduszu Zdrowia z dnia 19 grudnia, 2017; Załączniki nr 5 do zarządzenia Nr 125/2017/DGL Prezesa Narodowego Funduszu Zdrowia z dnia 19 grudnia, 2017; §22 pkt.1-3 zarządzenia Nr 125/2017/DGL Prezesa Narodowego Funduszu Zdrowia z dnia 19 grudnia, 2017).

Patients included in the programme receive drugs free of charge. In case of outpatient services a patient receives drugs directly from the hospital pharmacy, also free of charge. The provider controls treatment outcomes regularly and in case of lack of outcomes he is obliged to exclude the patient from the programme. The patients qualified into drug programme are regularly evaluated and undergo time-updated monitoring. In this area, the drug programme descriptions include criteria for exclusion from the programme. Fulfilling at least one of the listed exclusion criteria constitute the grounds for such actions (Załączniki nr 2 do zarządzenia Nr 125/2017/DGL Prezesa Narodowego Funduszu Zdrowia z dnia 19 grudnia, 2017; §8 zarządzenia Nr 125/2017/DGL Prezesa Narodowego Funduszu Zdrowia z dnia 19 grudnia, 2017; Załączniki nr 5 do zarządzenia Nr 125/2017/DGL Prezesa Narodowego Funduszu Zdrowia z dnia 19 grudnia, 2017; §22 pkt.1-3 zarządzenia Nr 125/2017/DGL Prezesa Narodowego Funduszu Zdrowia z dnia 19 grudnia, 2017).

It should be emphasized that drug programmes are an important mechanism of patients’ access to innovative, expensive, and mostly hospital based medical products. More and more often, oncology and non-oncology diseases resistant to standard therapeutic management require inclusion of new therapies, which often constitute the only and last treatment method available. At the same time, drug programmes with limited public funds, also enable control over reimbursement expenditure through precise definition of clinical criteria necessary to be met to include a patient into a programme.

Poland is the only country in the world where innovative, expensive, and mostly hospital based medical products are reimbursed under time-limited drug programmes, which is unique in world scale and it is not possible to compare the obtained results with other countries. The time limited drug programme mean that when patient meet the qualification criteria of this programme, he received treatment for ca. 2 years. In other countries, there are reimbursement limits, but they do not have strict inclusion and exclusion criteria.

## Aim of the Study

The primary aim of the study was to estimate public payer expenditures on drugs available within drug programmes from 2015-2018 in Poland. In addition, the goal was to analyse values of reimbursement realized by the NHF in terms of oncology and non-oncology drug programmes. Contract values reflect expected expenditures within a given year and are unable to compare total expenditures between drug programmes. This is particularly the case for programmes covering multiple medicinal products with more than one indication.

## Methods

### Subject of the Analysis

The study presents the National Health Fund expenditure on drug reimbursement used in drug programmes from 2015-2018. In addition, the total values of reimbursement realised by the NHF within drug programmes from 2015-2018 were divided into:

- Oncology drug programmes
- Non-oncology drug programmes

### Estimation – Amounts for Drug Reimbursement Used Within Drug Programmes

Estimation of cost for the reimbursement of medical products available within drug programmes was conducted based on data published by the Department of Drug Administration of the National Health Fund obtained using IKAR pro database from 2015 to 2018 (https://ikarpro.pl/en). The cost of treatment with active substance in a given drug program was calculated as the product of the number of refunded milligrams of the substance according to the data of the National Health Fund and the average real price determined based on messages from the Department of Drug Economy. Then the costs of all active substances were added together under the drug program.

Announcements on reimbursement value and volume (number of reimbursed unit packages) of drugs based on EAN identification code in Poland are published by the NHF each month (cumulative data). The NHF statements include total cost of drugs accounted for in drug programmes and in the “Chemotherapy” catalogue, without distinction on the amount spent on reimbursement of drugs financed under drug programmes and the amount spent on reimbursement of drugs available in the “Chemotherapy” catalogue.

The presentation of reimbursement values for medical products (according to name and active substance of a drug) required detailed analysis. Therefore, each EAN identification code had an assigned medical product name using Excel logical function in line with the list of reimbursed drugs published by the Ministry of Health.

### Estimation – Value of Agreements in the Range: Drug Programmes

Summary of reimbursement values realised by the NHF from 2015-2018, in terms of particular oncology and non-oncology drug programmes, were disclosed by the NHF.

Estimations were expressed as USD using the mean 2015-2018 exchange rate (1 USD 3.776): The weighted average exchanges rates were as follows: 1 USD in 2015 (3.7701), 2016 (3.9431), 2017 (3.777), and 2018 (3.6134) (http://www.nbp.pl/home.aspx?f=/kursy/arch_a.html).

## Results

During 2015 and 2016 respectively, 1,125,327 and 1,213,656 drug packages, representing 129–149 various brands and 110–118 various active substances, were available in drug programmes. Reimbursement of these products was associated with the NHF budget expenditure of more than 635–755 mln USD.

From 2017 and 2018 the number of actively available drug packages in drug programmes was 1,400,560 and 1,510,667, representing 169-182 various brands and 130-142 various active substances. Reimbursement of these products was associated with the NHF budget expenditure of approximately 854–921 mln USD. Detailed data are presented in **Table 1**.

**Table 1:** Amount spent on reimbursement of drugs used within drug programmes in Poland estimates for 2015-2018 [USD].

Year	2015 [USD]	2016 [USD]	2017 [USD]	2018 [USD]
**Drug Programmes**	635,434,206	755,656,672	854,272,086	921,165,770

Source: the authors based on NHF data.

### Reimbursement by Drug Programme Type From 2015-2018 in Poland

From 2015 to 2018 values of reimbursement under drug programmes were approximately 635–921 mln USD. Oncology drug programmes constituted 48.1–52.4% of these values and were approximately 305–483 mln USD. Whereas, values of non-oncology drug programmes were approximately 330–438 mln USD and constituted 51.9–47.6%. Until the end of 2018 the National Health Fund, under the contracts with the providers, financed 92 drug programmes (32 oncology and 60 non-oncology programmes). Detailed data were presented in **Table 2**.

**Table 2:** Reimbursement by drug programme type in years 2015-2018 in Poland [USD].

Year		2015	2016	2017	2018
**DRUG PROGRAMMES**	**Value [USD]**	**635,434,206**	**755,656,672**	**854,272,086**	**921,165,770**
	Number	69	73	83	92
**Oncological Drug Programmes**	**Value [USD]**	**305,367,160**	**321,264,182**	**402,520,374**	**483,023,398**
	Share [%]	48.1	42.5	47.1	52.4
	Number	49	52	29	32
**Nononcological Drug Programmes**	**Value [USD]**	**330,067,046**	**434,392,490**	**451,751,712**	**438,142,372**
	Share [%]	51.9	57.5	52.9	47.6
	Number	20	21	64	60

Source: the authors based on NHF data. Bolded volumes are the most important data in **Table 2**.

The highest value constituted contracts concluded in order to treat breast cancer, multiple sclerosis, chronic hepatitis C and kidney cancer. Appendices 1–3 present the list of drug programmes (oncology and non-oncology) realised by the providers during 2015–2018 along with summary value of the contract.

## Limitations

Important limitations during study preparation were that the information on reimbursement amount based on EAN codes combines data for drugs used in two reimbursement categories i.e chemotherapy and in drug programmes. Therefore, reimbursement for drugs used solely within drug programmes presented in **Tables 1** and **2** may be overestimated by approximately 5 to 10%.

There are more than 100 active substances currently financed by the NHF under drug programmes. Some of these active substances are financed in a number of various indications, hindering particular drug programmes cost calculation from published data. The majority of current drug programmes involve two or more substances.

The estimated amounts are closer to the real world payer expenditure on drugs due to inclusion of effective prices (with discounts), not listed. However, overall spending might be overestimated and it does not cover other confidential elements of risk sharing agreements, such as payback.

## Discussion and Results

The analysis focused on estimating public payer expenditure on drugs available within drug programmes during 2015–2018 in Poland and indication values of reimbursement realised by the NHF in terms of drug programmes divided into particular oncology and non-oncology drug programs. The research presented in this paper was based on analysis of data provided by the National Health Fund in Poland (NHF).

During 2015–2018 values of reimbursement under drug programmes were approximately 635–921 mln USD. Values of oncology drug programmes constituted 48.1–52.4% and were approximately 305–483 mln USD. Whereas values of non-oncology drug programmes were approximately 330–438 mln USD and constituted 51.9–47.6%.

Moreover, from 2015-2018 large increases in NHF expenses on drug programmes were observed. Until the end of 2018, the National Health Fund realized 92 drug programmes in the treatment of oncological and nononcological diseases, which was 23 more than in 2015.

Growing expenditure on health care, caused by demographic changes and an increase in civilisation diseases, among others, constitute currently one of the largest challenges for health care systems. Costs of oncological treatment are the highest and fastest growing health expenditure. Nonetheless, public expenditure on drugs, financed by the National Health Fund, both expressed in nominal value and in relation to GDP, in Poland is the lowest in Europe and among the lowest in OECD countries.

It should be emphasized, that in Poland, drug programmes are an important mechanism of patients’ access to innovative medical products. More and more often oncology and non-oncology diseases are resistant to standard therapeutic management and these patients require inclusion of new therapies, which often constitute the only and last treatment method available. At the same time, limited public funds drug programmes also enable control over reimbursement expenditure through precise definition of clinical criteria necessary to include a patient into a program.

Regarding this issue the risk-sharing instruments were presented in the Act of 12 May 2011 on the reimbursement of foodstuffs, drugs for particular nutritional uses and medical devices (hereinafter referred to as the Act on Reimbursement). Initiating risk-sharing agreements (RSA) for reimbursement decisions issued by the Minister of Health was a natural result of the necessity to address both patients’ growing needs, as well as payers’ limited financing capability. Simultaneously, the discussed solution is in alignment with the changes accepted in the drug return system, and enabled their formal implementation.

The Act on Reimbursement enables for a number of solutions that could be introduced as a risk sharing instrument. These could be based on, for example:

- having the applicant’s earnings be conditional on obtained health outcomes (outcome-based schemes).
- making the statutory ex-factory price conditional on the applicant’s provision of a lowered price for supplies such as a drug, foodstuff for particular nutritional uses and medical device, which was agreed upon during negotiations (rebates).
- making the statutory ex-factory price conditional on the sales volume of a drug, foodstuff for definite nutritional uses and medical device (price-volume agreement).
- making the statutory ex-factory price conditional on refunding a part of the obtained reimbursement to the public payer (payback schemes).
- determining other reimbursement options to help increase accessibility to guaranteed services or decrease the costs of these services.

The risk-sharing tools by way of the Act on Reimbursement were proposed to accomplish various goals, which are indicated in the Act (explanatory memorandum)

- providing access to new health technologies at a cost that is in alignment with the public payer’s capacity.
- expanding the range of available instruments associated with reduction of public healthcare costs through negotiations between the Economic Commission and potential applicants.
- restricting the negative effects of the reference pricing systems universally used by EU Member States, which involves comparisons of pharmaceutical prices between definite countries – prices in Poland are considered to be among the lowest in Europe – this represents applicants’ argument against further decreases of statutory prices, as that would cause a domino effect in terms of lowering prices in other countries, as well as a worsening of the situation connected to parallel export (RSA in Drugs Reimbursement System in Poland and Abroad; Ferrario et al., 2017).

It is anticipated that in the coming years, the increase in public spending on pharmaceuticals in the oncology sector will exceed the wider pharmaceutical market (Godman et al., 2019). As mentioned above, a significant number of innovative new therapies in oncology are expected to enter the market in the future. Many of them are expected to be breakthroughs in treatment in areas such as breast cancer and nonsmall cell lung cancer, significantly increasing survival. These agents are tested within many lines of therapy, as well as in combination with pre-existing and other new therapies (MS report). As a result, it is expected that the increase in public expenditure on oncological drugs by 2020 will exceed the total amount of expenses on reimbursement of medicines in general (MS report; Howard et al., 2015; Cohen, 2017).

Almost all large companies are currently producing oncological drugs or have these types of drugs as potential medicines in the coming years (Ringgaard Anne Science Nordic; EY Poland Access to innovative cancer drugs in Poland in comparison with selected European Union countries and Switzerland). Many new drugs, high-priced and facing little competition, are targeted at very specific types of cancer. Therefore, since biological treatments do not have the same general competition as chemical drugs, they have a longer shelf-life in terms of patent protection. The 20 best-selling anticancer drugs in the pharmaceutical industry generate annual sales exceeding $50 billion worldwide. Rituxan, Avastin, and Herceptin Roche lead the group, reaching $21 billion in sales for these three drugs (Top 20 Blockbuster Cancer Drugs). The best Roche oncology line accounted for approximately 40% of the top 20 in total sales. Detailed data were presented in **Table 3**. It is important to notice that our observations are very consistent with worldwide data. Within the top 20 products in Poland, is the same active substance, and the drug sale is driven by medical need in correlation with epidemiological indicators.

**Table 3:** Top 20 International blockbuster cancer drugs in 2018, [USD].

**Lp.**	**Brand Name**	**Active Substance**	**Manufacturer**	**Condition or Diseases Treated**	**Global Sales (USD)**
1	**Avastin**	Bevacizumab	Roche	Breast, colorectal, lung, kidney, ovarian cancers	6.7 bln
2	**Revlimid**	Lenalidomide	Celgene	Multiple myeloma	4.2 bln
3	**Rituxan**	Rituximab	Roche	Non-Hodgkins Lymphoma, chronic lymphocytic leukemia	7.5 bln
4	**Herceptin**	Trastuzumab	Roche	HER2+ breast cancer	6.5 bln
5	**Imbruvica**	Ibrutinib capsules	Johnson & Johnson/Pharmacyclics	Mantel cell lymphoma, chronic lymphocetic leukemia	5.3 bln
6	**Gleevec**	Imatinib	Novartis	Chronic myeloid leukemia, gastrointestinal stromal tumors	4.7 bln
7	**Alimta**	Pemetrexed	Eli Lilly	Nonsmall cell lung cancer	2.5 bln
8	**Velcade**	Bortezomib	Johnson & Johnson/Takeda	Multiple myeloma, mantle cell lymphoma	2.6 bln
9	**Erbitux**	Cetuximab	BMS/Merck Serono	Colorectal, head, and neck cancers	1.9 bln
10	**Gardasil**	Human Papillomavirus Quadrivalent (Types 6, 11, 16, and 18) Vaccine, Recombinant	Merck & Co.	Cervical cancer	1.8 bln
11	**Zytiga**	Abiraterone acetate	Johnson & Johnson	Prostate cancer	1.7 bln
12	**Xeloda**	Capecitabine	Roche	Breast, colorectal cancers	1.6 bln
13	**Tarceva**	Erlotinib	Roche	Nonsmall-cell lung, pancreatic cancers	1.4 bln
14	**Afinitor**	Everolimus	Novartis	Breast cancer	1.3 bln
15	**Tasigna**	Nilotinib	Novartis	Chronic myeloid leukemia	1.3 bln
16	**Sutent**	Sunitinib Malate	Pfizer	Renal cell sarcoma, gastrointestinal stromal tumors	1.2 bln
17	**Nexavar**	Sorafenib	Bayer	Renal cell sarcoma, liver cancer	1.0 bln
18	**Xgeva**	Denosumab	Amgen	Bone mestases	1.0 bln
19	**Zoladex**	Goserelin acetate	AstraZeneca	Breast, prostate cancer	1.0 bln
20	**Yervoy**	Ipilimumab	BMS	Melanoma	960 mln

Source: www.thebalance.com [access: 17.03.2018].

## Conclusion

1. In Poland, drug programmes cover treatment of oncological diseases and nononcological diseases and are an important mechanism of patients’ access to innovative, expensive, and mostly hospital based medical products.
2. There is a significant increase in NHF expenditures on drug programmes in Poland during 2015-2018.
3. The number of drug programmes increased from 69 in 2015 to 92 in 2018 covering more than 100 active substances. Increase was driven mainly by oncology programmes.

## Data Availability Statement

The datasets presented in this study can be found in online repositories. The names of the repository/repositories and accession number(s) can be found in the article/**Supplementary Material**.

## Author Contributions

AM participated in the study design, methods, collection of the data, quantitative analysis, and interpretation of data. ŁAP participated in quantitative analysis and interpretation of data. BD participated in the study design. MF-N discussed the study design. JJ participated in the study design, methods and collection of the data. WW participated in the interpretation of data. AS prepared the manuscript. JD participated in quantitative analysis and interpretation of data. AC participated in data collection. BJ participated in quantitative analysis and interpretation of data. MW participated in the study design and prepared the manuscript. MN participated in the study design, methods, quantitative analysis and interpretation of data. All authors contributed to the article and approved the submitted version.

## Conflict of Interest

The authors declare that the research was conducted in the absence of any commercial or financial relationships that could be construed as a potential conflict of interest.

## Abbreviations

NHF, National Health Fund; USD, United States Dollar.
