# Supplementary material for: Overview and Analysis of the Cost of Drug Programs in Poland: Public Payer Expenditures and Coverage of Cancer and Non-Neoplastic Diseases Related Drug Therapies from 2015–2018 Years
Source: Front Pharmacol. 2020 Aug 14;11:1123. doi: 10.3389/fphar.2020.01123 (PMC7456857; doi:10.3389/fphar.2020.01123)
Supplement: Supplementary file 1 [file DataSheet_1.pdf]

SUPPLEMENTARY DATA

REIMBURSEMENT BY DRUG PROGRAMME TYPE IN POLAND ESTIMATES TO 2015

Appendix. 1. Reimbursement by drug programme type in Poland estimates to 2015, [USD].

Lp.	Number of drug program	Name of drug program	Type	Values of reimbursement realised by the NHF, [USD]
**1**	B.9.	Treatment of breast cancer	oncological	78 485 573
**2**	B.29.	Treatment of multiple sclerosis	non-oncological	54 647 054
**3**	B.2.	Treatment of chronic viral hepatitis C	non-oncological	38 244 466
**4**	B.10.	Treatment of renal cell carcinoma	oncological	33 328 329
**5**	B.14.	Chronic myeloid leukemia	oncological	31 779 778
**6**	B.54.	Lenalidomide in the treatment of refractory or relapsed multiple myeloma	oncological	28 788 394
**7**	B.71.	Interferon-free treatment of chronic viral hepatitis C	non-oncological	28 727 888
**8**	B.4.	Treatment of advanced colorectal cancer	oncological	27 788 808
**9**	B.33.	Treatment of rheumatoid arthritis and aggressive juvenile idiopathic arthritis	non-oncological	27 346 493
**10**	B.3.	Treatment of gastrointestinal stromal tumours (GIST)	oncological	21 902 549
**11**	B.1.	Treatment of chronic viral hepatitis B	non-oncological	21 200 573
**12**	B.36.	Treatment of beneficiaries with severe active ankylosing spondylitis with TNF alfa inhibitors	non-oncological	18 834 410
**13**	B.31.	Treatment of pulmonary arterial hypertension (PAH)	non-oncological	18 695 466
**14**	B.59.	Treatment of melanoma of skin or mucosal melanoma	oncological	17 534 442
**15**	B.12.	Treatment of malignant lymphomas	oncological	16 893 316
**16**	B.48.	Treatment of skin melanoma	oncological	15 093 385
**17**	B.46.	Treatment of multiple sclerosis after first line treatment failure or rapidly evolving severe multiple sclerosis	non-oncological	12 716 300
**18**	B.23.	Treatment of Gaucher's disease	non-oncological	11 937 381

Lp.	Number of drug program	Name of drug program	Type	Values of reimbursement realised by the NHF, [USD]
**19**	B.6.	Treatment of non-small cell lung cancer	oncological	10 367 449
**20**	B.35.	Treatment of aggressive psoriatic arthritis	non-oncological	9 335 776
**21**	B.25.	Treatment of mucopolisacharosis type II (Hunter syndrome)	non-oncological	9 109 280
**22**	B.15.	Haemorrhages prevention in children with haemophilia A and B	non-oncological	8 863 718
**23**	B.22.	Treatment of Pompe disease	non-oncological	7 973 901
**24**	B.32.	Treatment of Crohn's disease (CD)	non-oncological	7 751 211
**25**	B.56.	Treatment of castration-resistant prostate cancer	oncological	7 642 649
**26**	B.39.	Treatment of secondary hyperparathyroidism in haemodialysis patients	non-oncological	7 641 937
**27**	B.40.	Prevention of respiratory syncytial virus infections	non-oncological	5 777 221
**28**	B.19.	Treatment of dwarf children with somatotropin pituitary insufficiency	non-oncological	5 688 023
**29**	B.44.	Treatment of severe ige-dependent allergic asthma with omalizumab	non-oncological	5 313 629
**30**	B.50.	Treatment of patients with advanced ovarian cancer	oncological	5 179 768
**31**	B.8.	Treatment of soft tissue sarcomas	oncological	3 674 907
**32**	B.47.	Treatment of moderate and severe plaque psoriasis	non-oncological	3 646 576
**33**	B.67.	Treatment with immunoglobulin transfusions in neurologic diseases	non-oncological	3 473 645
**34**	B.24.	Treatment of Hurler disease	non-oncological	2 898 332
**35**	B.5.	Treatment of hepatocellular carcinoma	oncological	2 525 210
**36**	B.62.	Treatment of primary immune deficiency (PID) in adult patients	non-oncological	2 361 726
**37**	B.28.	Treatment of focal dystonia and hemifacial spasm	non-oncological	2 140 716
**38**	B.17.	Treatment of primary immunodeficiency in children	non-oncological	2 023 049
**39**	B.26.	Treatment of mucopolysaccharidosis type IV (Maroteaux-Lamy syndrome)	non-oncological	1 756 214
**40**	B.58.	Treatment of advanced gastric cancer	oncological	1 483 182
**41**	B.70.	Treatment of neovascular (exudative) form of age-related macular degeneration (AMD)	non-oncological	1 220 918
**42**	B.53.	Treatment of well-differentiated neuroendocrine pancreatic carcinoma	oncological	1 163 269
**43**	B.20.	Treatment of dwarf children with severe primary igf-1 deficiency	non-oncological	1 065 371

Lp.	Number of drug program	Name of drug program	Type	Values of reimbursement realised by the NHF, [USD]
**44**	B.42.	Treatment of dwarf children with Turner syndrome (TS)	non-oncological	1 043 551
**45**	B.68.	Treatment of pulmonary arterial hypertension with sildenafil, epoprostenol and macitentan (PAH)	non-oncological	1 009 212
**46**	B.60.	Treatment of Gaucher's disease type 1	non-oncological	996 794
**47**	B.37.	Treatment of anaemia in the course of chronic renal insufficiency	non-oncological	917 417
**48**	B.55.	Treatment of patients with ulterative colitis	non-oncological	897 512
**49**	B.57.	Treatment of upper limb spasticity after stroke with botulinum toxin type A	non-oncological	877 637
**50**	B.65.	Treatment of Philadelphia chromosome-positive (Ph+) chronic acute lymphoblastic leukemia with dasatinib	oncological	794 456
**51**	B.30.	Treatment of spasticity in cerebral palsy	non-oncological	780 659
**52**	B.27.	Treatment of chronic lung infections in beneficiaries with mucoviscidosis	non-oncological	650 177
**53**	B.69.	Treatment with paricalcitol of secondary hyperparathyroidism in haemodialysis patients	non-oncological	512 270
**54**	B.49.	Oral treatment of iron overload states in the body	non-oncological	511 029
**55**	B.66.	Treatment with bexarotene of mycosis fungoides or Sézary syndrome	non-oncological	427 935
**56**	B.18.	Treatment of premature sexual maturation in children	non-oncological	367 710
**57**	B.52.	Treatment of squamous cell cancer of head and neck in combination with radition therapy in locally advanced disease	oncological	365 720
**58**	B.72.	Treatment of melanoma with combination therapy with dabrafenib and trametinib	oncological	315 583
**59**	B.63.	Treatment of non-small cell lung cancer with afatinib	oncological	260 392
**60**	B.43.	Treatment of viral hepatitis B in beneficiaries after transplantations or in beneficiaries with lymphomas, treated with rituximab	non-oncological	208 648
**61**	B.41.	Treatment of Prader-Willi syndrome	non-oncological	177 211
**62**	B.38.	Treatment of dwarf children with chronic kidney failure	non-oncological	109 261
**63**	B.64.	Treatment with growth hormone of dwarf children born too small in relation to pregnancy length (sga or iugr)	non-oncological	57 541
**64**	B.21.	Treatment of severe familial hyperhomocysteinemia	non-oncological	50 563
**65**	B.75.	Treatment of active granulomatosis with polyangiitis (gpa) or microscopic polyangiitis (mpa)	non-oncological	34 340
**66**	B.74.	Treatment of chronic thromboembolic pulmonary hypertension (cteph)	non-oncological	27 247
**67**	B.73.	Treatment of neurogenic detrusor overactivity	non-oncological	10 860

Lp.	Number of drug program	Name of drug program	Type	Values of reimbursement realised by the NHF, [USD]
**68**	B.61.	Treatment of nephropathic juvenile cystinosis	non-oncological	8 197
**69**	B.76.	Treatment of tyrosinemia type 1 (ht-1)	non-oncological	0

Source: own elaboration, NHF data.

REIMBURSEMENT BY DRUG PROGRAMME TYPE IN POLAND ESTIMATES TO 2016

Appendix. 2. Reimbursement by drug programme type in Poland estimates to 2016, [USD].

Lp.	Number of drug program	Name of drug program	Type	Values of reimbursement realised by the NHF, [USD]
**1**	B.71.	Interferon-free treatment of chronic viral hepatitis C	non-oncological	116 043 837
**2**	B.9.	Treatment of breast cancer	oncological	82 626 134
**3**	B.29.	Treatment of multiple sclerosis	non-oncological	58 685 457
**4**	B.10.	Treatment of renal cell carcinoma	oncological	34 788 764
**5**	B.33.	Treatment of rheumatoid arthritis and aggressive juvenile idiopathic arthritis	non-oncological	32 656 860
**6**	B.54.	Lenalidomide in the treatment of refractory or relapsed multiple myeloma	oncological	31 718 703
**7**	B.4.	Treatment of advanced colorectal cancer	oncological	31 532 468
**8**	B.14.	Chronic myeloid leukemia	oncological	31 292 976
**9**	B.3.	Treatment of gastrointestinal stromal tumours (GIST)	oncological	23 254 701
**10**	B.36.	Treatment of beneficiaries with severe active ankylosing spondylitis with TNF alfa inhibitors	non-oncological	19 035 602
**11**	B.1.	Treatment of chronic viral hepatitis B	non-oncological	18 998 771
**12**	B.31.	Treatment of pulmonary arterial hypertension (PAH)	non-oncological	17 609 026
**13**	B.12.	Treatment of malignant lymphomas	oncological	17 211 421
**14**	B.70.	Treatment of neovascular (exudative) form of age-related macular degeneration (AMD)	non-oncological	16 524 091
**15**	B.46.	Treatment of multiple sclerosis after first line treatment failure or rapidly evolving severe multiple sclerosis	non-oncological	16 387 806

Lp.	Number of drug program	Name of drug program	Type	Values of reimbursement realised by the NHF, [USD]
**16**	B.23.	Treatment of Gaucher's disease	non-oncological	12 247 283
**17**	B.48.	Treatment of skin melanoma	oncological	12 086 553
**18**	B.59.	Treatment of melanoma of skin or mucosal melanoma	oncological	11 564 784
**19**	B.67.	Treatment with immunoglobulin transfusions in neurologic diseases	non-oncological	10 933 407
**20**	B.15.	Haemorrhages prevention in children with haemophilia A and B	non-oncological	10 751 162
**21**	B.56.	Treatment of castration-resistant prostate cancer	oncological	9 582 437
**22**	B.35.	Treatment of aggressive psoriatic arthritis	non-oncological	9 240 550
**23**	B.6.	Treatment of non-small cell lung cancer	oncological	8 596 134
**24**	B.22.	Treatment of Pompe disease	non-oncological	8 261 478
**25**	B.25.	Treatment of mucopolisacharosis type II (Hunter syndrome)	non-oncological	8 119 287
**26**	B.68.	Treatment of pulmonary arterial hypertension with sildenafil, epoprostenol and macitentan (PAH)	non-oncological	7 314 161
**27**	B.72.	Treatment of skin cancer with dabrafenib	oncological	7 254 412
**28**	B.32.	Treatment of Crohn’s disease (CD)	non-oncological	7 151 874
**29**	B.50.	Treatment of patients with advanced ovarian cancer	oncological	6 341 163
**30**	B.19.	Treatment of dwarf children with somatotropin pituitary insufficiency	non-oncological	5 974 292
**31**	B.44.	Treatment of severe ige-dependent allergic asthma with omalizumab	non-oncological	5 785 056
**32**	B.39.	Treatment of secondary hyperparathyroidism in haemodialysis patients	non-oncological	5 719 989
**33**	B.40.	Prevention of respiratory syncytial virus infections	non-oncological	5 717 411
**34**	B.62.	Treatment of primary immune deficiency (PID) in adult patients	non-oncological	5 268 223
**35**	B.2.	Treatment of chronic viral hepatitis C	non-oncological	5 110 817
**36**	B.8.	Treatment of soft tissue sarcomas	oncological	4 140 049
**37**	B.47.	Treatment of moderate and severe plaque psoriasis	non-oncological	4 128 566
**38**	B.5.	Treatment of hepatocellular carcinoma	oncological	3 257 092
**39**	B.24.	Treatment of Hurler disease	non-oncological	3 044 951
**40**	B.77.	Treatment of resistant and relapse lymphomas cd30+	non-oncological	2 789 296
**41**	B.69.	Treatment with paricalcitol of secondary hyperparathyroidism in haemodialysis patients	non-oncological	2 301 767

Lp.	Number of drug program	Name of drug program	Type	Values of reimbursement realised by the NHF, [USD]
**42**	B.28.	Treatment of focal dystonia and hemifacial spasm	non-oncological	2 273 806
**43**	B.17.	Treatment of primary immunodeficiency in children	non-oncological	2 072 179
**44**	B.74.	Treatment of chronic thromboembolic pulmonary hypertension (cteph)	non-oncological	1 913 521
**45**	B.58.	Treatment of advanced gastric cancer	oncological	1 726 773
**46**	B.63.	Treatment of non-small cell lung cancer with afatinib	oncological	1 345 313
**47**	B.53.	Treatment of well-differentiated neuroendocrine pancreatic carcinoma	oncological	1 273 548
**48**	B.65.	Treatment of Philadelphia chromosome-positive (Ph+) chronic acute lymphoblastic leukemia with dasatinib	oncological	1 108 758
**49**	B.60.	Treatment of Gaucher's disease type 1	non-oncological	1 080 163
**50**	B.42.	Treatment of dwarf children with Turner syndrome (TS)	non-oncological	1 073 089
**51**	B.20.	Treatment of dwarf children with severe primary igf-1 deficiency	non-oncological	1 064 833
**52**	B.57.	Treatment of upper limb spasticity after stroke with botulinum toxin type A	non-oncological	963 595
**53**	B.76.	Treatment of tyrosinemia type 1 (ht-1)	non-oncological	938 053
**54**	B.26.	Treatment of mucopolysaccharidosis type IV (Maroteaux-Lamy syndrome)	non-oncological	925 455
**55**	B.30.	Treatment of spasticity in cerebral palsy	non-oncological	852 478
**56**	B.37.	Treatment of anaemia in the course of chronic renal insufficiency	non-oncological	802 289
**57**	B.66.	Treatment with bexarotene of mycosis fungoides or Sézary syndrome	non-oncological	764 950
**58**	B.27.	Treatment of chronic lung infections in beneficiaries with mucoviscidosis	non-oncological	743 424
**59**	B.55.	Remission induction in ulcerative colitis	non-oncological	704 633
**60**	B.49.	Oral treatment of iron overload states in the body	non-oncological	615 274
**61**	B.52.	Treatment of squamous cell cancer of head and neck in combination with radiation therapy in locally advanced disease	oncological	520 243
**62**	B.18.	Treatment of premature sexual maturation in children	non-oncological	430 970
**63**	B.75.	Treatment of active granulomatosis with polyangiitis (gpa) or microscopic polyangiitis (mpa)	non-oncological	429 808
**64**	B.43.	Treatment of viral hepatitis B in beneficiaries after transplantations or in beneficiaries with lymphomas, treated with rituximab	non-oncological	218 676
**65**	B.41.	Treatment of children with Prader-Willi syndrome	non-oncological	188 621
**66**	B.64.	Treatment with growth hormone of dwarf children born too small in relation to pregnancy length (sga lub	non-oncological	174 771

Lp.	Number of drug program	Name of drug program	Type	Values of reimbursement realised by the NHF, [USD]
		iugr)		
**67**	B.79.	Treatment of chronic lymphocytic leukemia with obinutuzumab	non-oncological	127 420
**68**	B.38.	Treatment of dwarf children with chronic kidney failure	non-oncological	90 410
**69**	B.21.	Treatment of severe familial hyperhomocysteinemia	non-oncological	55 864
**70**	B.73.	Treatment of neurogenic detrusor overactivity	non-oncological	45 940
**71**	B.80.	Maintenance therapy with olaparib of patients with relapsed platinum-sensitive advanced ovarian cancer, fallopian tube cancer or primary peritoneal cancer	oncological	41 756
**72**	B.61.	Treatment of nephropathic juvenile cystinosis	non-oncological	21 130
**73**	B.78.	Treatment of primary immune deficiencies in adult patients with normal human immunoglobulin administered with human recombined hyaluronidase	non-oncological	20 118

Source: own elaboration, NHF data.

REIMBURSEMENT BY DRUG PROGRAMME TYPE IN POLAND ESTIMATES TO 2017

Appendix. 3. Reimbursement by drug programme type in Poland estimates to 2017, [USD].

Lp.	Number of drug program	Name of drug program	Type	Values of reimbursement realised by the NHF, [USD]
**1**	B.9.	Treatment of breast cancer	oncological	118 276 084
**2**	B.71.	Interferon-free treatment of chronic viral hepatitis C	non-oncological	109 178 658
**3**	B.29.	Treatment of multiple sclerosis	non-oncological	66 378 300
**4**	B.54.	Treatment of refractory or relapsed multiple myeloma	oncological	36 985 168
**5**	B.10.	Treatment of renal cell carcinoma	oncological	35 019 759
**6**	B.4.	Treatment of advanced colorectal cancer	oncological	34 302 745

Lp.	Number of drug program	Name of drug program	Type	Values of reimbursement realised by the NHF, [USD]
**7**	B.33.	Treatment of rheumatoid arthritis and aggressive juvenile idiopathic arthritis	non-oncological	33 284 884
**8**	B.14.	Treatment of chronic myeloid leukemia	oncological	28 998 737
**9**	B.59.	Treatment of skin or mucosal melanoma	oncological	23 710 717
**10**	B.3.	Treatment of gastrointestinal stromal tumours (GIST)	oncological	21 657 287
**11**	B.36.	Treatment of beneficiaries with severe active ankylosing spondylitis with TNF alfa inhibitors	non-oncological	19 046 874
**12**	B.46.	Treatment of multiple sclerosis after first line treatment failure or rapidly evolving severe multiple sclerosis	non-oncological	18 823 968
**13**	B.31.	Treatment of pulmonary arterial hypertension (PAH)	non-oncological	18 700 871
**14**	B.70.	Treatment of neovascular (exudative) form of age-related macular degeneration (AMD)	non-oncological	18 103 475
**15**	B.12.	Treatment of malignant lymphomas	oncological	16 949 679
**16**	B.1.	Treatment of chronic viral hepatitis B	non-oncological	16 726 079
**17**	B.67.	Treatment with immunoglobulin transfusions in neurologic diseases	non-oncological	14 663 919
**18**	B.72.	Treatment of melanoma with combination therapy with dabrafenib and trametinib	oncological	14 174 447
**19**	B.56.	Treatment of castration-resistant prostate cancer	oncological	13 387 547
**20**	B.23.	Treatment of Gaucher’s disease type 1	non-oncological	13 357 659
**21**	B.15.	Haemorrhages prevention in children with haemophilia A and B	non-oncological	11 488 207
**22**	B.68.	Treatment of pulmonary arterial hypertension with sildenafil, epoprostenol and macitentan (PAH)	non-oncological	10 770 605
**23**	B.35.	Treatment of aggressive psoriatic arthritis	non-oncological	10 314 324
**24**	B.22.	Treatment of Pompe disease	non-oncological	9 579 385
**25**	B.77.	Treatment of resistant and relapse lymphomas cd30+	oncological	9 355 582

Lp.	Number of drug program	Name of drug program	Type	Values of reimbursement realised by the NHF, [USD]
**26**	B.32.	Treatment of Crohn’s disease (CD)	non-oncological	8 454 469
**27**	B.48.	Treatment of skin melanoma	oncological	8 210 473
**28**	B.6.	Treatment of non-small cell lung cancer	oncological	7 614 113
**29**	B.50.	Treatment of patients with advanced ovarian cancer	oncological	7 409 942
**30**	B.44.	Treatment of severe ige-dependent allergic asthma with omalizumab	non-oncological	6 782 211
**31**	B.40.	Prevention of respiratory syncytial virus infections	non-oncological	6 562 042
**32**	B.25.	Treatment of mucopolisacharosis type II (Hunter syndrome)	non-oncological	6 506 251
**33**	B.19.	Treatment of dwarf children with somatotropin pituitary insufficiency	non-oncological	6 401 367
**34**	B.62.	Treatment of primary immunodeficiency in adults	non-oncological	5 757 874
**35**	B.47.	Treatment of moderate and severe plaque psoriasis	non-oncological	5 223 983
**36**	B.8.	Treatment of soft tissue sarcomas	oncological	4 288 962
**37**	B.39.	Treatment of secondary hyperparathyroidism in haemodialysis patients	non-oncological	3 950 574
**38**	B.74.	Treatment of chronic thromboembolic pulmonary hypertension (cteph)	non-oncological	3 298 036
**39**	B.5.	Treatment of hepatocellular carcinoma	oncological	3 283 906
**40**	B.79.	Treatment of chronic lymphocytic leukemia with obinutuzumab	oncological	3 071 287
**41**	B.24.	Treatment of Hurler disease	non-oncological	2 935 966
**42**	B.63.	Treatment of non-small cell lung cancer with afatinib and nintedanib	oncological	2 468 621
**43**	B.28.	Treatment of focal dystonia and hemifacial spasm	non-oncological	2 392 192
**44**	B.81.	Treatment of primary mielofibrosis and secondary mielofibrosis in the course of polycyhemia and thrombocythosis	oncological	2 382 702

Lp.	Number of drug program	Name of drug program	Type	Values of reimbursement realised by the NHF, [USD]
**45**	B.80.	Maintenance therapy with olaparib of patients with relapsed platinum-sensitive advanced ovarian cancer, fallopian tube cancer or primary peritoneal cancer	oncological	2 329 554
**46**	B.17.	Treatment of primary immunodeficiency in children	non-oncological	2 290 480
**47**	B.2.	Treatment of chronic viral hepatitis c	non-oncological	2 198 818
**48**	B.58.	Treatment of advanced gastric cancer	oncological	2 097 652
**49**	B.55.	Treatment of patients with colitis ulcerosis	non-oncological	1 837 395
**50**	B.53.	Treatment of well-differentiated neuroendocrine pancreatic carcinoma	oncological	1 766 397
**51**	B.26.	Treatment of mucopolysaccharidosis type iv (maroteaux-lamy syndrome)	non-oncological	1 728 893
**52**	B.87.	Treatment of idiopatic pulmonary fibrosis	non-oncological	1 671 679
**53**	B.84.	Lenalidomid for the treatment of patients with anemia depending on transformations in the course of mylodysplastic assemblies with low or indirect risk, risk associated with cytogenetic irregularity with 5q deletion	oncological	1 586 077
**54**	B.69.	Treatment with paricalcitol of secondary hyperparathyroidism in haemodialysis patients	non-oncological	1 529 137
**55**	B.76.	Treatment of tyrosinemia type 1 (ht-1)	non-oncological	1 499 983
**56**	B.78.	Treatment of primary immune deficiencies in adult patients with normal human immunoglobulin administered with human recombined hyaluronidase	non-oncological	1 389 103
**57**	B.60.	Treatment of gaucher's disease type 1	non-oncological	1 244 807
**58**	B.42.	Treatment of dwarf children with turner syndrome (ts)	non-oncological	1 032 587
**59**	B.20.	Treatment of dwarf children with severe primary igf-1 deficiency	non-oncological	887 751
**60**	B.27.	Treatment of chronic lung infections in beneficiaries with mucoviscidosis	non-oncological	832 382
**61**	B.65.	Treatment of acute lymphoblastic leukemia	oncological	826 749
**62**	B.30.	Treatment of spasticity in cerebral palsy	non-oncological	810 558

Lp.	Number of drug program	Name of drug program	Type	Values of reimbursement realised by the NHF, [USD]
**63**	B.37.	Treatment of anaemia in the course of chronic renal insufficiency	non-oncological	778 431
**64**	B.57.	Treatment of upper limb spasticity after stroke with botulinum toxin type a	non-oncological	762 256
**65**	B.66.	Treatment with bexarotene of mycosis fungoides or sézary syndrome	oncological	736 061
**66**	B.88.	Treatment of patients with advanced basocellular skin cancer wismodegib	oncological	609 818
**67**	B.85.	Treatment of patients with metastatic pancreatic adenocarcinoma	oncological	603 611
**68**	B.18.	Treatment of premature sexual maturation in children	non-oncological	478 466
**69**	B.75.	Treatment of active granulomatosis with polyangiitis (gpa) or microscopic polyangiitis (mpa)	non-oncological	475 941
**70**	B.64.	Treatment with growth hormone of dwarf children born too small in relation to pregnancy length (sga or iugr)	non-oncological	409 770
**71**	B.49.	Oral treatment of iron overload states in the body	non-oncological	372 776
**72**	B.52.	Treatment of squamous cell cancer of head and neck	oncological	364 229
**73**	B.41.	Treatment of prader-willi syndrome	non-oncological	207 472
**74**	B.43.	Treatment of viral hepatitis b in beneficiaries after transplantations or in beneficiaries with lymphomas, treated with rituximab	non-oncological	186 964
**75**	B.38.	Treatment of dwarf children with chronic kidney failure	non-oncological	114 339
**76**	B.83.	Treatment of lower limb spasticity after stroke with botulinum toxin type a	non-oncological	89 713
**77**	B.82.	Treatment of certolizumab pegol patients with heavy, active form of axial spondyloartropathy (spa) without radiographic changes	non-oncological	69 770
**78**	B.21.	Treatment of severe familial hyperhomocysteinemia	non-oncological	63 785
**79**	B.89.	Treatment with ewerolimus of patients with sclerosis tuberosa non-qualifying for operative treatments with *subependymal* giant cells (sega)	oncological	52 467
**80**	B.61.	Treatment of nephropathic juvenile cystinosis	non-oncological	39 084
**81**	B.86.	Treatment of congenitated auto-inflammatory assemblies	non-oncological	36 356
**82**	B.90.	Treatment of motor disorders in the course of advanced parkinson's disease	non-oncological	17 303

Lp.	Number of drug program	Name of drug program	Type	Values of reimbursement realised by the NHF, [USD]
**83**	B.73.	Treatment of neurogenic detrusor overactivity	non-oncological	13 543

Source: own elaboration, NHF data.

REIMBURSEMENT BY DRUG PROGRAMME TYPE IN POLAND ESTIMATES TO 2018

Appendix. 4. Reimbursement by drug programme type in Poland estimates to 2018, [USD].

Lp.	Number of drug program	Name of drug program	Type	Values of reimbursement realised by the NHF, [USD]
**1**	B.9.	Treatment of breast cancer	oncological	120 564 937
**2**	B.29.	Treatment of multiple sclerosis	non-oncological	74 570 625
**3**	B.71.	Interferon-free treatment of chronic viral hepatitis C	non-oncological	58 957 833
**4**	B.4.	Treatment of advanced colorectal cancer	oncological	46 374 846
**5**	B.54.	treatment of refractory or relapsed multiple myeloma	oncological	43 222 355
**6**	B.10.	Treatment of renal cell carcinoma	oncological	38 155 171
**7**	B.33.	Treatment of rheumatoid arthritis and aggressive juvenile idiopathic arthritis	non-oncological	35 534 988
**8**	B.59.	Treatment of skin or mucosal melanoma	oncological	32 643 073
**9**	B.14.	Treatment of chronic myeloid leukemia	oncological	29 714 120
**10**	B.31.	Treatment of pulmonary arterial hypertension (PAH)	non-oncological	26 660 996
**11**	B.56.	Treatment of castration-resistant prostate cancer	oncological	22 327 920
**12**	B.3.	Treatment of gastrointestinal stromal tumours (GIST)	oncological	22 273 244

Lp.	Number of drug program	Name of drug program	Type	Values of reimbursement realised by the NHF, [USD]
**13**	B.70.	Treatment of neovascular (exudative) form of age-related macular degeneration (AMD)	non-oncological	21 045 965
**14**	B.46.	Treatment of multiple sclerosis after first line treatment failure or rapidly evolving severe multiple sclerosis	non-oncological	20 021 314
**15**	B.36.	Treatment of beneficiaries with severe active ankylosing spondylitis with TNF alfa inhibitors	non-oncological	19 835 842
**16**	B.72.	Treatment of melanoma with combination therapy with dabrafenib and trametinib	oncological	18 463 676
**17**	B.6.	Treatment of non-small cell lung cancer	oncological	17 049 924
**18**	B.12.	Treatment of malignant lymphomas	oncological	15 579 222
**19**	B.67.	Treatment with immunoglobulin transfusions in neurologic diseases	non-oncological	15 333 074
**20**	B.23.	Treatment of Gaucher's disease	non-oncological	14 673 701
**21**	B.35.	Treatment of aggressive psoriatic arthritis	non-oncological	11 102 138
**22**	B.68.	Treatment of pulmonary arterial hypertension with sildenafil, epoprostenol and macitentan (pah)	non-oncological	10 909 480
**23**	B.32.	Treatment of crohn's disease (cd)	non-oncological	9 712 364
**24**	B.81.	Treatment of primary mielofibrosis and secondary mielofibrosis in the course of polycyhemia and thrombocythosis	oncological	9 323 293
**25**	B.87.	Treatment of idiopathic pulmonary fibrosis	non-oncological	9 265 419
**26**	B.15.	Haemorrhages prevention in children with haemophilia a and b	non-oncological	8 739 265
**27**	B.1.	Treatment of chronic viral hepatitis b	non-oncological	8 684 694
**28**	B.48.	Treatment of skin melanoma	oncological	8 626 239
**29**	B.22.	Treatment of pompe disease	non-oncological	8 567 666
**30**	B.77.	Treatment of resistant and relapse lymphomas cd30+	oncological	7 935 812
**31**	B.80.	Maintenance therapy with olaparib of patients with relapsed platinum-sensitive advanced ovarian cancer,	oncological	7 755 700

Lp.	Number of drug program	Name of drug program	Type	Values of reimbursement realised by the NHF, [USD]
		fallopian tube cancer or primary peritoneal cancer		
**32**	B.50.	Treatment of patients with advanced ovarian cancer	oncological	7 391 638
**33**	B.40.	Prevention of respiratory syncytial virus infections	non-oncological	7 306 999
**34**	B.44.	Treatment of severe ige-dependent allergic asthma with omalizumab	non-oncological	6 977 968
**35**	B.19.	Treatment of dwarf children with somatotropin pituitary insufficiency	non-oncological	6 869 004
**36**	B.25.	Treatment of mucopolisacharosis type ii (hunter syndrome)	non-oncological	6 678 216
**37**	B.47.	Treatment of aggressive psoriatic arthritis	non-oncological	6 112 470
**38**	B.62.	Treatment of primary immunodeficiency in adults	non-oncological	5 549 987
**39**	B.79.	Treatment of chronic lymphocytic leukemia with obinutuzumab	oncological	5 514 203
**40**	B.74.	Treatment of chronic thromboembolic pulmonary hypertension (cteph)	non-oncological	4 589 063
**41**	B.84.	Lenalidomid for the treatment of patients with anemia depending on transformations in the course of mylodysplastic assemblies with low or indirect risk, risk associated with cytogenetic irregularity with 5q deletion	oncological	3 914 369
**42**	B.92.	Ibrutynib in the treatment of patients with chronic lymphocytic leukemia	oncological	3 768 442
**43**	B.8.	Treatment of soft tissue sarcomas	oncological	3 478 333
**44**	B.88.	Treatment of patients with advanced basocellular skin cancer wismodegib	oncological	3 438 567
**45**	B.39.	Treatment of secondary hyperparathyroidism in haemodialysis patients	non-oncological	3 218 567
**46**	B.63.	Treatment of non-small cell lung cancer with afatinib and nintedanib	oncological	3 076 843
**47**	B.95.	Treatment of atypical hemolytic-uremic syndrome (*ahus*)	non-oncological	3 048 972
**48**	B.96.	Treatment of paroxysmal nocturnal hemoglobinuria	non-oncological	3 022 699
**49**	B.5.	Treatment of hepatocellular carcinoma	oncological	2 855 103

Lp.	Number of drug program	Name of drug program	Type	Values of reimbursement realised by the NHF, [USD]
**50**	B.78.	Treatment of primary immune deficiencies in adult patients with normal human immunoglobulin administered with human recombined hyaluronidase	non-oncological	2 852 819
**51**	B.24.	Treatment of Hurler disease	non-oncological	2 836 066
**52**	B.17.	Treatment of primary immunodeficiency in children	non-oncological	2 832 343
**53**	B.55.	Treatment of patients with ulterative colitis	non-oncological	2 781 703
**54**	B.28.	Treatment of focal dystonia and hemifacial spasm	non-oncological	2 578 618
**55**	B.85.	Treatment of patients with metastatic pancreatic adenocarcinoma	oncological	2 329 798
**56**	B.58.	Treatment of advanced gastric cancer	oncological	2 262 861
**57**	B.53.	Treatment of well-differentiated neuroendocrine pancreatic carcinoma	oncological	1 731 430
**58**	B.69.	Treatment of secondary hyperparathyroidism in haemodialysis patients	non-oncological	1 513 055
**59**	B.60.	Treatment of gaucher's disease type i	non-oncological	1 422 153
**60**	B.26.	Treatment of mucopolysaccharidosis type IV (Maroteaux-Lamy syndrome)	non-oncological	1 334 243
**61**	B.76.	Treatment of tyrosinemia type 1 (ht-1)	non-oncological	1 271 372
**62**	B.20.	Treatment of dwarf children with severe primary igf-1 deficiency	non-oncological	1 135 330
**63**	B.42.	Treatment of dwarf children with Turner syndrome (TS)	non-oncological	1 059 483
**64**	B.2.	Treatment of chronic viral hepatitis C	non-oncological	1 057 169
**65**	B.27.	Treatment of chronic lung infections in beneficiaries with mucoviscidosis	non-oncological	885 183
**66**	B.57.	Treatment of upper limb spasticity after stroke with botulinum toxin type a	non-oncological	867 516
**67**	B.65.	Treatment of acute lymphoblastic leukemia	oncological	849 380
**68**	B.30.	Treatment of spasticity in cerebral palsy	non-oncological	786 463

Lp.	Number of drug program	Name of drug program	Type	Values of reimbursement realised by the NHF, [USD]
**69**	B.89.	Treatment with ewerolimus of patients with sclerosis tuberosa non-qualifying for operative treatments with *subependymal* giant cells (sega)	oncological	772 851
**70**	B.64.	Treatment with growth hormone of dwarf children born too small in relation to pregnancy length (sga or iugr)	non-oncological	749 597
**71**	B.37.	Treatment of anaemia in the course of chronic renal insufficiency	non-oncological	731 097
**72**	B.66.	Treatment with beksarotene of mycosis fungoides or sézary syndrome	oncological	718 945
**73**	B.90.	Treatment of motor disorders in the course of advanced parkinson's disease	non-oncological	611 303
**74**	B.75.	Treatment of active granulomatosis with polyangiitis (gpa) or microscopic polyangiitis (mpa)	non-oncological	523 936
**75**	B.82.	Treatment of certolizumab pegol patients with heavy, active form of axial spondyloartropathy without radiographic changes	non-oncological	509 266
**76**	B.18.	Treatment of premature sexual maturation in children	non-oncological	507 553
**77**	B.83.	Treatment of lower limb spasticity after stroke with botulinum toxin type a	non-oncological	450 700
**78**	B.93.	Pixsantron for the treatment of malignant lymphoma	oncological	427 712
**79**	B.97.	Treatment of adults with idiopathic thrombocytopenic purpura	non-oncological	352 674
**80**	B.100.	Treatment of resistant and recurrental form of hodgkin's classic lymphoma using niwolumab	oncological	259 071
**81**	B.86.	Treatment of congenitated auto-inflammatory disorders	non-oncological	243 684
**82**	B.52.	Treatment of squamous cell cancer of head and neck	oncological	224 321
**83**	B.41.	Treatment of prader - willi syndrome	non-oncological	220 229
**84**	B.49.	Oral treatment of iron overload states in the body	non-oncological	219 311
**85**	B.98.	Treatment of pediatric patients with chronic primary immuno thrombocythopenia	non-oncological	215 894
**86**	B.43.	Treatment of viral hepatitis B in beneficiaries after transplantations or in beneficiaries with lymphomas, treated with rituximab	non-oncological	170 902
**87**	B.91.	Treatment of severe immunoresistant pemphigus with rituximab	non-oncological	144 466
**88**	B.99.	Treatment of acromegal with passyothide	non-oncological	103 823
**89**	B.38.	Treatment of dwarf children with chronic kidney failure	non-oncological	102 225

Lp.	Number of drug program	Name of drug program	Type	Values of reimbursement realised by the NHF, [USD]
**90**	B.21.	Treatment of severe familial hyperhomocysteinemia	non-oncological	54 227
**91**	B.61.	Treatment of nephropathic juvenile cystinosis	non-oncological	18 842
**92**	B.73.	Treatment of neurogenic detrusor overactivity	non-oncological	9 815

Source: own elaboration, NHF data.
